# Gas Transport in Glassy Polymers: Prediction of Diffusional Time Lag

**DOI:** 10.3390/membranes8010008

**Published:** 2018-02-03

**Authors:** Matteo Minelli, Giulio C. Sarti

**Affiliations:** Department of Civil, Chemical, Environmental and Materials Engineering (DICAM), Alma Mater Studiorum-University of Bologna, Via Terracini, 28, 40131 Bologna, Italy; matteo.minelli@unibo.it

**Keywords:** gas permeability, diffusion, thermodynamics, NELF model, glassy polymers

## Abstract

The transport of gases in glassy polymeric membranes has been analyzed by means of a fundamental approach based on the nonequilibrium thermodynamic model for glassy polymers (NET-GP) that considers the penetrant chemical potential gradient as the actual driving force of the diffusional process. The diffusivity of a penetrant is thus described as the product of a purely kinetic quantity, the penetrant mobility, and a thermodynamic factor, accounting for the chemical potential dependence on its concentration in the polymer. The NET-GP approach, and the nonequilibrium lattice fluid (NELF) model in particular, describes the thermodynamic behavior of penetrant/polymer mixtures in the glassy state, at each pressure or composition. Moreover, the mobility is considered to follow a simple exponential dependence on penetrant concentration, as typically observed experimentally, using only two adjustable parameters, the infinite dilution penetrant mobility *L*_10_ and the plasticization factor *β*, both determined from the analysis of the dependence of steady state permeability on upstream pressure. The available literature data of diffusional time lag as a function of penetrant upstream pressure has been reviewed and compared with model predictions, obtained after the values of the two model parameters (*L*_10_ and *β*), have been conveniently determined from steady state permeability data. The model is shown to be able to describe very accurately the experimental time lag behaviors for all penetrant/polymer pairs inspected, including those presenting an increasing permeability with increasing upstream pressure. The model is thus more appropriate than the one based on Dual Mode Sorption, which usually provides an unsatisfactory description of time lag and required an ad hoc modification.

## 1. Introduction

The analysis of the solubility and the transport properties of gases and vapors in polymers has a practical utility in various industrial applications, such as gas separation and purification [1,2], ion exchange [3,4], biomedical [5], sensing [6] and packaging operations [7]. For this reason, much effort has been devoted to the experimental characterization of the permeability of solutes in polymeric systems, and in glassy polymers in particular, accounting for both solubility and diffusional properties. 

The transport of low molecular weight species in dense polymeric films is associated to two separate contributions, according to the solution-diffusion mechanism [8], and thus the steady state permeability of penetrant 1 is given by:

(1) $$P_{1}=\frac{J_{1,ss}}{p_{1}^{u}-p_{1}^{d}}\frac{l}{M_{1}}=<S_{1}>\cdot <D_{1}>$$

The permeability *P*_1_ is given by the product of average solubility coefficient $<S_{1}>$ and average diffusivity, $<D_{1}>$, respectively. In Equation (1), *J*_1,*ss*_ is the steady state diffusive mass flux of penetrant 1, *M*_1_ is its molecular mass, *l* is the membrane thickness, while $p_{1}^{u}$ and $p_{1}^{d}$ indicate upstream and downstream penetrant partial pressures, respectively. Clearly, when $p_{1}^{d}$ is negligibly small $<S_{1}>$ in Equation (1) becomes simply the upstream value of the solubility coefficient.

Steady state permeation experiments readily provide the value of penetrant permeability in the operating conditions inspected, while the analysis of the transient permeation process allows the determination of the average diffusion coefficient through the diffusional time lag ($\tau_{L}$) method [9]:

(2) $$\tau_{L}=\frac{l^{2}}{6<D_{1}>}$$

Equation (2) has been obtained directly from the solution of the transient mass balance equation, in the case of negligible permeate concentration on the downstream side of the membrane [10]. Consequently, the simultaneous evaluation of $P_{1}$, $<D_{1}>$, and $<S_{1}>$ from the same transient permeation experiment is often obtained, based also on Equation (1).

Besides the experimental investigation, the modeling description of such systems is of great importance for the fundamental understanding of the physical mechanisms involved, as well as for practical design purposes aiming at both the development of novel membranes and at the optimization of the process operating conditions.

The modeling description of gas solubility and transport in glassy polymers is still frequently performed by using the Dual Mode Sorption (DMS) approach [11,12], developed more than 50 years ago, based on the assumption that one can differentiate two separate populations of penetrant molecules, one dissolved within the polymer matrix and one adsorbed onto the surface of the micro-voids supposed to be present in the glassy phase [13]. As far as mass transport is concerned, the DMS model considers separate Fickian diffusions of the two populations, with two constant values for the diffusivities of the dissolved and adsorbed species, *D*_D_ and *D*_H_, respectively; their values eventually determine the overall rate of transport in the glassy phase, based on local mass balance and accounting for possible mechanisms of exchanges between the two populations. Two possible mechanisms have been mainly considered to that aim, total immobilization [14] and partial immobilization [15] schemes, which lead to somewhat elaborate but explicit expressions for penetrant steady state permeability and diffusional time lag. Further modifications have also been proposed, e.g., considering Langmuir kinetics for adsorption and desorption as exchange mechanisms between the two populations [16], rarely used likely in view of the increased number of adjustable parameters required. Recently, Wang et al. proposed the so-called dual diffusion model (DDM), a different limiting variant of the DMS model [17], specifically developed in order to improve the representation of time lag data, which are in general not well described by the other different DMS based approaches. In such a limiting model, the two populations considered by the DMS approach diffuse independently in the matrix and are not subject to any mutual interaction. Thus, local equilibrium or exchange mechanism between dissolved and adsorbed populations are not allowed, so that the molecule that enters as adsorbed is mobile but will remain always adsorbed, and the molecule that enters as dissolved is mobile but will remain always dissolved; i.e., the transport of the two populations occurs through separate pathways, as if one could conceive the dense region and the micro-voids as both continuous and fully interconnected phases.

Moreover, several severe drawbacks are associated to the dual mode model both for the representation of solubility as well as of transport behaviors, as the DMS model is not able to represent all possible sorption and permeation behaviors encountered experimentally. Indeed, the description of S-shaped solubility isotherms encountered for example in alkyl alcohols sorption [18,19] is out of reach for the DMS model. On the other hand, it can only represent permeability behaviors that decrease with increasing upstream pressure, while the increasing [20] or nonmonotonous permeability trends [21] cannot be even qualitatively described by the DSM model so that such behaviors are considered due to a new phenomenon, the so-called *plasticization,* introduced to make the DSM model consistent with experimental behaviors.

In this work, the transport of low molecular weight species in glassy polymers, and more specifically the behavior of the diffusion time lag, is analyzed by means of a thermodynamic based approach recently proposed. The model considers the glassy polymer as a nonequilibrium homogeneous phase in which the penetrants are dissolved so that the gradient of the penetrant chemical potential is the driving force of the diffusive flux, as usual [22]; thus, it describes the diffusion coefficient as the product of the mobility coefficient, a purely kinetic quantity, and a thermodynamic factor, accounting for the concentration dependence of the penetrant chemical potential in the nonequilibrium glassy phase [23]. The approach relies on the nonequilibrium thermodynamics for glassy polymers (NET-GP) for the description of the thermodynamic behavior of penetrant/polymer mixtures in the glassy state, here required for the calculation of penetrant solubility and thermodynamic factor [24,25]. Remarkably, the model is readily obtained from the fundamentals of solute transport in homogeneous phases, considering a single population of the penetrant species.

Such an approach, which will be indicated as Standard Transport model for Glassy Polymers (STM-GP), has been already successfully employed to describe CO_2_ permeability as a function of upstream pressure in a variety of glassy polymers, showing all kinds of behaviors, including increasing and nonmonotonous trends [23], as well as in blends, copolymers [26] or in semicrystalline polymers [27], and even in high free volume glassy polymers [28,29]; the model proved to be effective also for light gases [30] or vapors [31]. Interestingly, the two model parameters, *L*_10_ and *β*, have been correlated with the properties of the pure polymer and pure penetrant, thus leading to the derivation of a simple procedure for the prediction of gas permeability in glassy polymeric membranes [32]. The STM-GP approach has been recently extended also to the case of multicomponent diffusion of a gaseous binary mixture in glassy polymers [33].

This work is specifically dedicated to the analysis of transient permeation, and the STM-GP model is here used to predict the diffusional time lag at different upstream pressures for a variety of solutes in different glassy polymers as bisphenol a polycarbonate (PC), polyvinyl chloride (PVC) and poly(ethylmethacrylate) (PEMA), by using the parameters determined from independent steady-state permeability data, already analyzed in previous papers [23,30]. The results obtained are then compared with the experimental data reported in the technical literature, showing that the model not only is fundamentally based and simpler to use than the different versions of the DMS model, but it is also accurate to describe and to predict time lag data in a straightforward way for all penetrant/polymer pairs. The same model formulation is useful to represent both steady state and transient transport data of gaseous penetrant in glassy polymers, and it is also applicable for the cases in which permeability increases with upstream pressure.

## 2. Theoretical Background

The STM-GP model and the nonequilibrium thermodynamic approach (NET-GP and NELF) require the calculation of penetrant solubility in glassy polymers, as described in detail in a previous work [23]. Thus, only the main features are here recalled, for the sake of simplicity. The use of such models for the calculation of diffusional time lag is then discussed.

### 2.1. NELF Model

The transport model relies on the thermodynamic description provided by the NET-GP approach, suitable to represent the properties of nonequilibrium glassy phases, and to calculate the solubility of gases [34], vapors [35] and liquids [36] in any kind of glassy polymers [37,38,39]. 

The model considers the actual value of the polymer density, *ρ*_2_, as a state variable to be used in addition to the usual set (*T*, *p* and composition), in order to describe the behavior of a nonequilibrium glassy phase. Therefore, the value of polymer density, different from that of the corresponding equilibrium state, measures the departure of the system from equilibrium conditions. The main result of the NET-GP approach is that the nonequilibrium Helmholtz free energy density of the penetrant/polymer mixture in the glassy phase can be calculated from the corresponding equilibrium expression, at the same temperature *T*, composition *ω*_1_, and at the same polymer density *ρ*_2_ prevailing in the glassy phase:

(3) $$a^{NE}\left(T,p,\rho_{2}^{},\omega_{1}\right)=a^{Eq}\left(T,\rho_{2}^{},\omega_{1}\right)$$

Thus, the NET-GP approach provides an extension to the glassy state of conventional equilibrium equation of state (EoS) approaches, such as the lattice fluid (LF) by Sanchez and Lacombe [40] or the statistical associating fluid theory [41,42]; based on that, any EoS which is appropriate for polymeric mixtures can be used for the derivation of the corresponding nonequilibrium models [43].

In this work, the STM-GP model considers the lattice fluid EoS by Sanchez and Lacombe, used in combination with the corresponding nonequilibrium theory (NELF model); thus, it makes use of three characteristic parameters for each pure species, either penetrant or polymer, for the representation of the thermodynamic properties, namely, the characteristic temperature T*, characteristic pressure p* and close-packed density ρ*. Their values have been retrieved from the literature, and the values used for the systems considered hereafter are listed in Table 1.

The lattice fluid EoS model uses appropriate mixing rules in which a dimensionless binary interaction parameter *k*_12_ enters for the description of the mixture properties [47]; the values of *k*_12_ can be determined from the analysis of vapor-liquid equilibrium (VLE) data for the equilibrium rubbery phases, when available, or from nonequilibrium solubility by the NELF model. The binary interaction parameters for each penetrant/polymer pair used in this work have been already determined in previous works, and their values are listed in Table 2.

Finally, the NELF model requires the actual polymer density, as input information for the determination of the penetrant solubility, accounting for the fact that *ρ*_2_ varies in general with penetrant pressure. To that aim, in the lack of direct polymer dilation measurement, the rheological model by Minelli and Doghieri [48,49] has been considered, taking advantage of the fact that in a broad range of penetrant pressure all data available indicate a linear relationship between penetrant pressure and polymer specific volume; therefore:

(4) $$\frac{1}{\rho_{2}^{}\left(p_{1}\right)}=\frac{1+k_{sw}p_{1}}{\rho_{2}^{0}}$$

The values of swelling coefficient *k_sw_* calculated by means of the rheological model by Minelli and Doghieri are also included in Table 2.

### 2.2. Transport Model

The penetrant permeability in glassy polymers is described by means of the STM-GP approach already illustrated in a previous work [23]. The model follows the fundamental treatment of binary mixtures, which considers the negative gradient of penetrant chemical potential as the driving force for diffusion [23]. Thus, the diffusion coefficient *D*_1_ is expressed by the following relationship:

(5) $$D_{1}=\alpha_{1}\cdot L_{1}=\frac{\partial \mu_{1}/RT}{\partial \ln\omega_{1}}\cdot L_{1}$$

The diffusion coefficient *D*_1_ is thus the product of two contributions:

(i) the thermodynamic factor *α*_1_;
(ii) the mobility coefficient *L*_1_, which is a purely kinetic quantity.

The first contribution is readily calculated by the NELF model for polymer glasses, which provides an explicit expression for the penetrant chemical potential. The mobility coefficient depends on the penetrant mass fraction in the membrane, *ω*_1_; although a general dependence could be used, a simple exponential relationship proved sufficient for most practical purposes, so that we consider [23]:

(6) $$L_{1}=L_{10}\cdot \exp\left(\beta \;\omega_{1}\right)$$

The two parameters *L*_10_ and *β*, the infinite dilution mobility coefficient and the plasticization factor, respectively, are determined from the analysis of experimental steady state permeability data. The first one accounts for the effects of penetrant size and polymer free volume, whereas the latter is associated to the membrane swellability in the presence of the diffusing species [32].

Based on Equations (5) and (6) and on the definition of permeability, a simple expression can be derived for the penetrant permeability through a glassy polymeric membrane whose upstream and downstream sides are at penetrant partial pressures $p_{1}^{u}$ and $p_{1}^{d}$, respectively, corresponding to penetrant solubilities $\omega_{1}^{u}$ and $\omega_{1}^{d}$; one has [23]:

(7) $$P_{1}=\frac{\rho_{20}\;L_{10}}{M_{1}\left(p_{1}^{u}-p_{1}^{d}\right)}\int_{p_{1}^{d}}^{p_{1}^{u}}\exp\left(\beta \omega_{1}\right)z_{1}\;\frac{\omega_{1}}{p_{1}}\;dp_{1}$$

in which *M*_1_ and *z*_1_ are the molecular weight and the compressibility factor of the penetrant; the latter can be evaluated by a suitable equation of state (e.g., Peng Robinson). The solubility coefficient $S_{1}=\frac{\omega_{1}}{p_{1}}$ is calculated by the NELF model, which provides the required correlation between penetrant pressure in the gas phase and the corresponding mass fraction in the glassy polymeric membrane.

**Figure 1:**
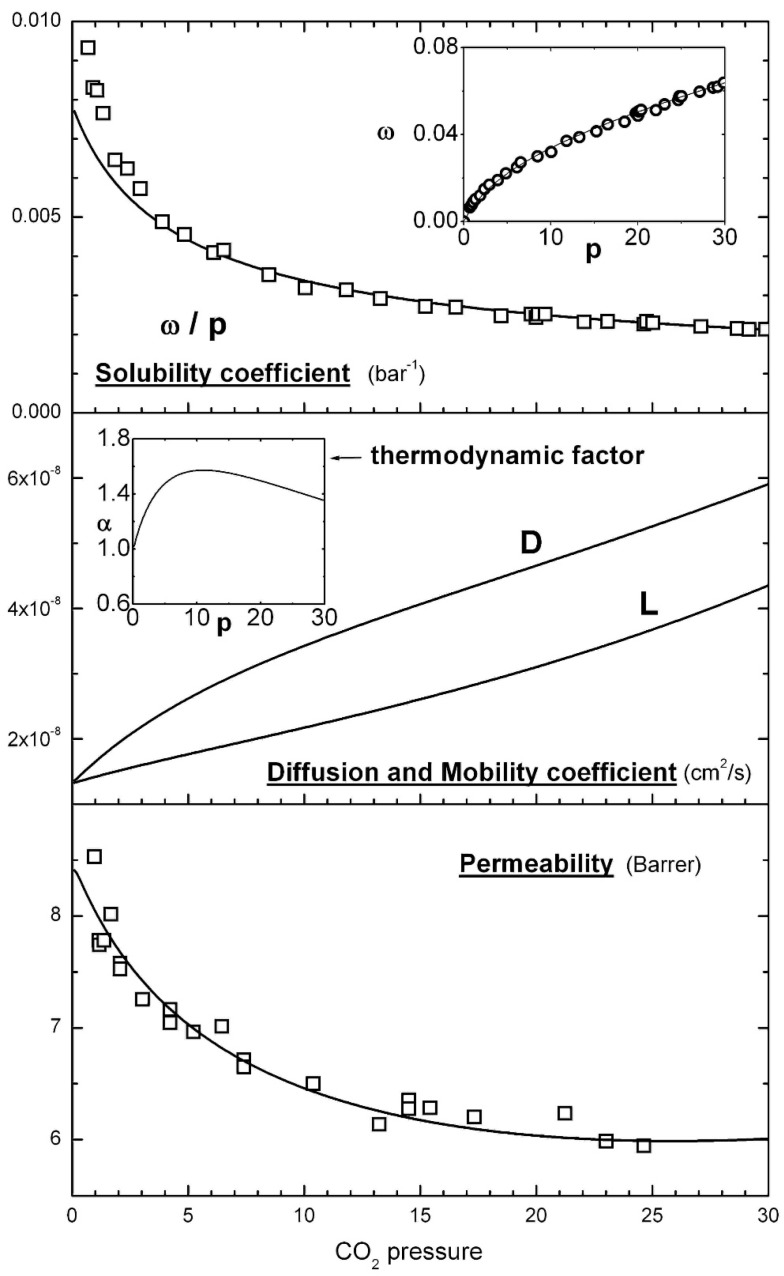
Illustration of the NELF/STM-GP model for the solubility and permeability of gases in a glassy polymer; in the example, the experimental data reported by Koros et al. [50] of CO_2_ in glassy polycarbonate (PC) at 35 °C are reported together with the model curves [23].

The features of the thermodynamic models for solubility and transport are schematically illustrated in Figure 1, which reports the analysis of the solubility and permeability data by Koros et al. [50] already carried in a previous work [23,30].

The transport parameters *L*_10_ and *β* for the penetrant/polymer couples considered have been already determined from the analysis of the steady state permeability isotherms reported in previous works [23,30]. Their values are listed in Table 3 and will be employed for the time lag calculations at various upstream pressures.

### 2.3. Diffusional Time Lag

The time lag of diffusion, $\tau_{L}$, is defined as the intercept on the time axis of the asymptotic line representing the steady state trend of the overall permeated mass. Its value is typically obtained experimentally from the analysis of transient permeation data, and may also be calculated from the modelling of the same process; clearly, it provides a good estimate of the average diffusion coefficient [9]. For the model calculations, the expression of penetrant diffusive mass flux:

(8) $$J_{1}=-\rho L_{1}\omega_{1}\frac{\partial }{\partial x}\left(\frac{\mu_{1}}{RT}\right)$$

is used to obtain the amount of penetrant mass *Q*(*t*) permeated per unit area through the downstream surface of the membrane (located at *x* = *l*), over the entire time span between time zero and time *t*; one thus has:

(9) $$Q\left(t\right)=\int_{0}^{t}{J_{1}|}_{x=l}\;d\tau =-\int_{0}^{t}{\rho L_{1}\omega_{1}\frac{\partial }{\partial x}\left(\frac{\mu_{1}}{RT}\right)|}_{x=l}d\tau$$

**Figure 2:**
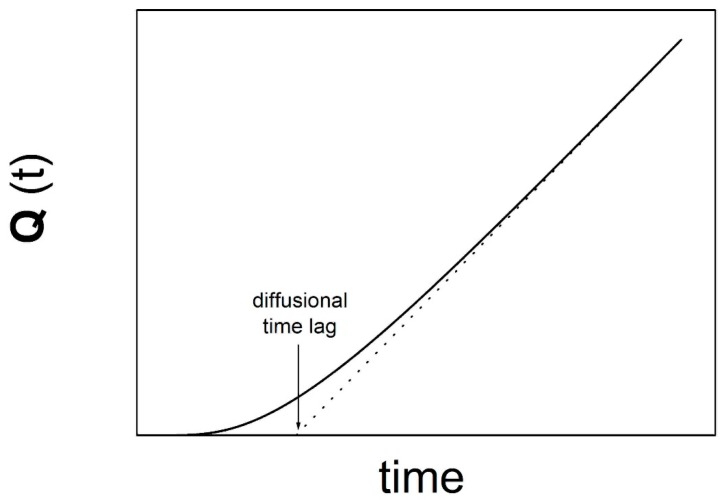
Illustration of the graphical determination of the diffusional time lag from the permeated mass per unit area vs. time plot.

The time dependence of *Q*(*t*) shows an upward curvature at early times (transient permeation), while it reaches a linear behavior when steady state conditions are achieved. The typical behavior is reported in Figure 2, in which the graphical determination of the diffusional time lag is apparent. 

For the case of constant diffusion coefficient, an explicit expression for the permeate mass per unit area *Q*(*t*) exists, and Equation (2) can be easily obtained [9]. However, penetrant diffusion coefficient may significantly depend on its concentration within the polymer matrix, and also the solubility coefficient may be (and often is) a clear function of penetrant pressure. Consequently, the time lag values for permeation in glassy polymers are appreciably affected by the upstream pressure applied to the membrane, as it was shown experimentally [51,52,53]. 

In this work, the diffusional time lag has been calculated numerically, following a three step procedure:

(i) the problem of transient one-dimensional penetrant diffusion in a glassy polymeric membrane is solved numerically (finite elements method), considering the appropriate boundary conditions (for each upstream penetrant pressure) given by the solubility values provided by the NELF model, and using the local mobility values calculated from Equation (6);
(ii) from the results obtained at each upstream pressure, the penetrant mass permeated per unit area *Q*(*t*) is calculated at any time according to Equation (9);
(iii) the time lag is determined from the intercept on the time axis of the steady state line asymptotically reached by the quantity *Q*(*t*).

The calculations are rather straightforward and their details are here omitted for simplicity sake.

In general, the model is applied to describe penetrant sorption and transport in glassy polymers through the following steps:

(a) the solubility curve (penetrant concentration vs. pressure) is first inspected and described by the NELF model, which makes use of polymer density, as well as values of swelling (*k*_sw_) and binary interaction (*k*_12_) coefficients; when not available, the last two parameters are retrieved from the analysis of the experimental data;
(b) steady state transport data (penetrant permeability vs. upstream pressure) is analyzed and modeled, using the solubility coefficient dependence of penetrant pressure provided by the NELF model, thus retrieving the parameters *L*_10_ and *β*;
(c) transient transport properties (diffusional time lag vs. upstream pressure) are finally calculated in a predictive fashion, making use of the description of penetrant solubility and mobility obtained, with no additional parameters.

## 3. Results

A selection of the literature time lag data obtained from transient permeation experiments has been analyzed in this section, as function of the penetrant upstream pressure, and compared with the predictions resulting from the STM-GP model calculations. For the sake of completeness the curves obtained by Dual Mode Sorption (both total [14] and partial immobilization [15] schemes), and DDM [17] models are also included in the plots for the sake of comparison.

### 3.1. Gas Transport in Glassy Bisphenol a Polycarbonate (PC)

**Figure 3:**
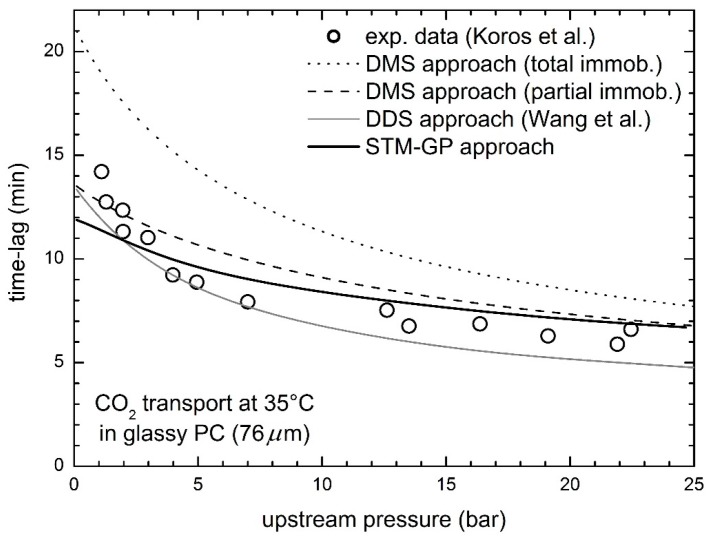
Diffusional time lag for CO_2_ transport in glassy PC at 35 °C: experimental data by Koros et al. [50] and STM-GP model predictions.

Steady state transport of CO_2_ in glassy PC experimentally investigated by Koros et al. [50] was already discussed in a previous work [23]; here we analyze its time lag behavior. The experimental permeation data (Figure 3) reveal a marked decreasing trend of the time lag as function of the upstream pressure with an upward curvature, clearly indicating an enhancement of the diffusion coefficient at increasing CO_2_ pressure, i.e., as the concentration within the polymer increases. 

As one can see, the STM-GP model provides a very good description of the experimental data based on the parameters already available, obtained from independent experiments, with only minor differences mostly in the range of very low penetrant pressures. Conversely, the DMS model overestimates significantly the time lag values in the whole range, with poorer prediction for the total immobilization (TI) than for the partial immobilization (PI) version. The behavior predicted by the Dual Diffusion Model, specifically developed for a better description of the time lag, is appreciably below the experimental data, although the qualitative trends are very similar.

**Figure 4:**
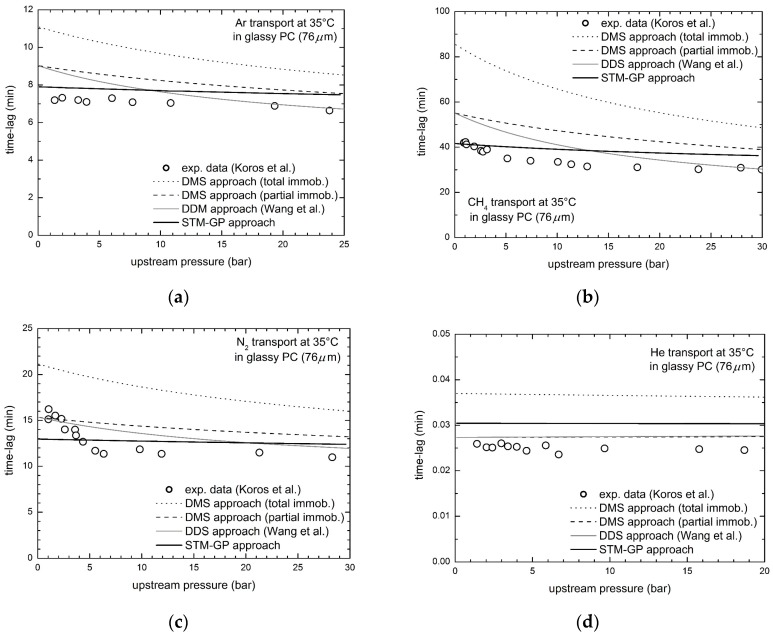
Diffusional time lag for gas transport in glassy PC at 35 °C, experimental data by Koros et al. [51] and STM-GP model predictions: (**a**) Ar; (**b**) CH_4_; (**c**) N_2_; and (**d**) He.

The analysis of the transport behavior of other relevant gaseous penetrants (Ar, N_2_, CH_4_ and He) in the same PC membranes has been carried out by Koros et al. [51]. The results in terms of time lag as function of upstream pressure are reported in Figure 4, and compared with the model predictions.

All four penetrants show decreasing behaviors of the time lag with upstream pressure, although with very different pressure sensitivity: it is appreciably declining for CH_4_, similarly to the CO_2_ case, whereas a slighter dependence is observed for N_2_ and Ar, while He time lag is approximately constant. Interestingly, the present STM-GP model is able to provide a satisfactory prediction of the experimental data for all the penetrants, even though some deviations can be clearly detected. However, a slightly better agreement between model and experimental data is observed for Ar, N_2_ and CH_4_ (Figure 4a–c) with respect to DMS and DDM approaches, while poorer description is obtained only for the case of He (Figure 4d). 

For a proper comparison, the relative average errors, *ε_ave_*, between experimental time lag data and model calculations are reported in Table 4 for each penetrant/polymer pair, for our STM-GP model as well as for all models considered; the relative average error is defined as:

(10) $$\varepsilon_{ave}=\frac{1}{n}\sum_{1}^{n}\frac{\left|\tau_{L,i}^{\exp.}-\tau_{L,i}^{calc.}\right|}{\tau_{L,i}^{\exp.}}$$

As one can see, a satisfactory description of the transient permeation data in PC is obtained by the STM-GP model with errors around 10% for all penetrants but He. The other approaches are not able to represent the time lag data equally well, and appreciably higher errors are obtained for DMS based models, both TI and PI, while DDM performs similarly to STM-GP model, for the PC cases inspected. It is worthwhile to recall, however, that DDM, specifically developed to better describe time-lag data, is inapplicable to PEMA/CO_2_ case and, in addition, it represents a very limiting situation that ignores local mass balance conditions and any exchange mechanism between the two diffusing populations.

### 3.2. Gas Transport in Glassy PVC

**Figure 5:**
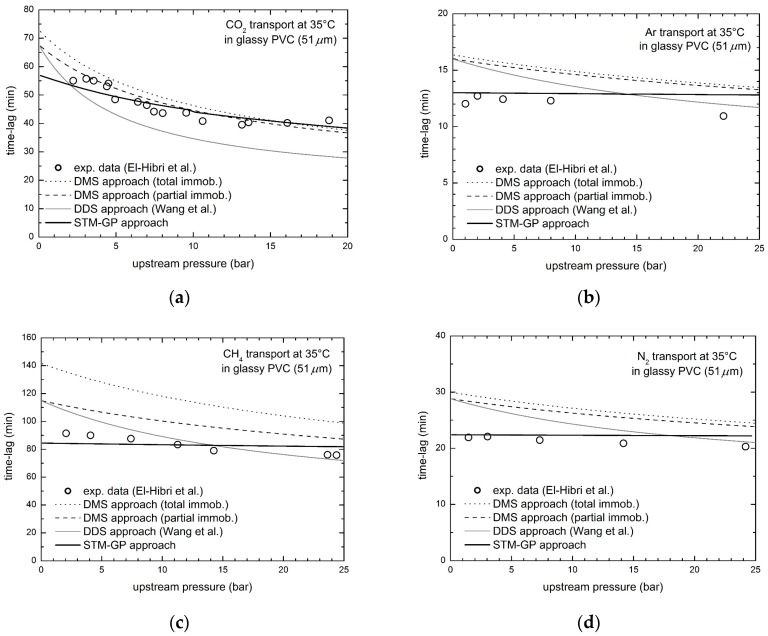
Diffusional time lag for gas transport in glassy PVC at 35 °C, experimental data by El-Hibri et al. [52] and STM-GP model predictions: (**a**) CO_2_; (**b**) Ar; (**c**) CH_4_; and (**d**) N_2_.

Solubility and steady state permeability data in PVC, presented by El-Hibri et al. [52], have been already analyzed in a previous work [30] for all the penetrants, thus obtaining the relevant transport parameters indicated in Table 3. The time lag behaviors of the four gases are now considered and reported in Figure 5 as function of penetrant upstream pressure, in the range 0–25 atm. Figure 5 includes also the predictions provided by the STM-GP model, as well as by DMS and DDM approaches for the sake of comparison. 

The time lag of CO_2_ transport is significantly decreasing as penetrant upstream pressure increases, while that of CH_4_ is only slightly declining and, on the contrary, those of Ar and N_2_ are practically constant. The STM-GP transport model predicts well the experimental results in the whole pressure range inspected; the trend predicted is in a remarkably good agreement with CO_2_ data, and also with the almost constant behaviors for the other penetrants, based on the values of the transport parameters obtained from independent experimental data. As one can see in Figure 5, the present model is significantly more effective in the representation of the experimental data, as DMS and DDM approaches overestimate time lag values and predict a more significant dependence on penetrant pressure. On the contrary, only minor deviations are observed between our transport model and the experimental data in PVC. Indeed, relative average errors are in the orders of 5–6%, well below those obtained by the other DMS based approaches (see Table 4).

### 3.3. Gas Transport in Glassy Poly(Ethylmethacrylate) (PEMA)

Chiou and Paul experimentally investigated solubility and transport of the different gases in glassy PEMA [53], revealing behaviors quite different from the ones considered previously, and in contrast with the typical trends well described by the DMS model. Indeed, the N_2_ solubility is linear with pressure in the range inspected and, consequently, the parameters of the alleged Langmuir populations (C’_H_, *b* and *D*_H_) cannot be determined or are practically zero. Furthermore, the CO_2_ steady state permeability in PEMA shows a strong increasing trend with upstream pressure, definitely in contrast with the DMS model description, so that such a behavior was considered due to a new phenomenon, indicated as the so-called plasticization. On the contrary, the STM-GP approach here adopted can be used in general to describe the behavior of all penetrants in all glassy polymers, with no need of any further assumptions specific for particular penetrant/polymer pairs, and no need to invoke the onset of a new phenomenon. Therefore, such framework can be used to describe any kind of gas permeability data, irrespective of their dependence on pressure, either decreasing, or increasing or also non-monotonous.

**Figure 6:**
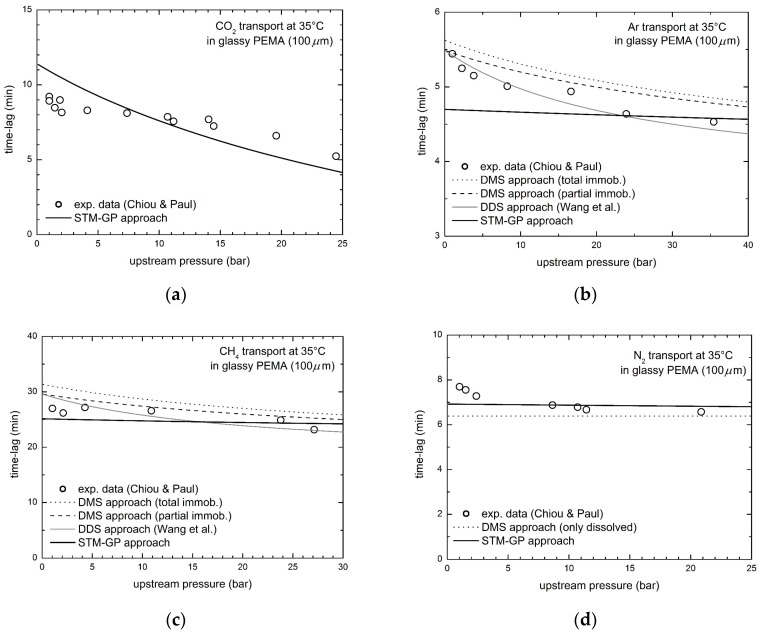
Diffusional time lag for gas transport in glassy PEMA at 35 °C, experimental data by Chiou and Paul [53] and STM-GP model predictions: (**a**) CO_2_; (**b**) Ar; (**c**) CH_4_; and (**d**) N_2_.

Figure 6 reports the diffusional time lag behavior of CO_2_, CH_4_, Ar and N_2_ in PEMA as a function of upstream penetrant pressure measured by Chiou and Paul [53], together with the transport model predictions. The curves obtained by the DMS and DDM models are also included for the sake of comparison, except for the case of CO_2_, which is out of reach by those models, as already discussed, in view of the increasing trend of steady state permeability with pressure. Noteworthy, in the case of N_2_ transport, the DMS and DDM models collapse on the same expression, as in the experimental sorption data no contribution to the overall N_2_ solubility can be attributed to the adsorbed population [53].

Remarkably, the STM-GP approach provides a quite good description of the time lag behaviors, with some little deviations between experimental data and model predictions, especially for the case of Ar diffusion, for which the DDM approach seems to be better (see the relative average errors in Table 4). The case of CO_2_ transport, however, cannot be described by the dual mode based models, DDM included, due to the increasing permeability behavior, while on the contrary it is well predicted by our present model, simply using values of the model parameters obtained from independent experimental data.

It is important to stress that the STM-GP model provides an accurate description of both steady state and transient permeation data for all penetrant/polymer pairs, as it does not face any intrinsic limitation requiring the introduction of a new phenomenon (as the so-called plasticization effect).

## 4. Conclusions

The transport of low molecular weight species in glassy polymeric membranes has been thoroughly investigated, and the dependence of the diffusional time lag on penetrant upstream pressure has been specifically assessed and predicted. The thermodynamic-based STM-GP model has been applied for the prediction of the time lag of diffusion by using the values of the two model parameters already obtained from independent experimental data as steady state permeability isotherms. The results were compared with literature experimental data of transient permeation, as well as with the results obtained from different models based on the dual mode sorption approach.

The STM-GP model considered in this work uses only two parameters for the description of the mobility coefficient (*L*_10_ and *β*) and proved already very effective in representing all kinds of permeability dependence on penetrant upstream pressure, including increasing or nonmonotonous trends. The comparison of the experimental time lag data with the values calculated in a predictive way by the STM-GP model confirm the ability of the model to describe and predict rather satisfactorily the different aspects of mass transport behavior in glassy polymeric membranes, consistently with its solid fundamental bases. The predicted time lag behaviors are indeed very close to the experimental data with only minor deviations, often appreciably smaller than the deviations obtained by DMS based models and even by the DDM model, which considers a limiting situation specifically developed to improve the description of time lag. The features observed for gas transport in glassy polymers are indeed all well described by the STM-GP model, either when there is a significant time lag decrease with upstream pressure, as observed for CO_2_, or when there is a much weaker dependence on pressure as observed for lighter gases such as N_2_ or Ar.

It is also noteworthy that our transport model is general in character and can be used for the description of any kind of permeability behavior (e.g., either decreasing or increasing or nonmonotonous with increasing upstream pressure), since it does not face any intrinsic limitation. Thus, both permeability and time lag behaviors of CO_2_ in PEMA, for which permeability increases with upstream pressure, can be well described by our model, while they are completely out of reach by the other DMS based approaches. Furthermore, the STM-GP approach is also simple in its nature, as it does not need to account for different populations of the penetrant diffusing species, and the diffusion mechanism is simply based on the general fundamentals of thermodynamics and mass transport. 

## Figures and Tables

**Table 1:** Characteristic parameters of the lattice fluid EoS/nonequilibrium LF model.

Species	T* (K)	p* (MPa)	ρ* (g/cm^3^)	Ref.
CO_2_	300	630	1.515	[24]
N_2_	145	160	0.943	[44]
Ar	190	180	1.400	[45]
CH_4_	215	250	0.500	[44]
He	9.3	4	0.148	[45]
PC	755	534	1.275	[24]
PVC	680	620	1.487	[30]
PEMA	602	568	1.221	[46]

**Table 2:** Binary interaction parameters *k*_12_ for the Sanchez and Lacombe EoS and nonequilibrium swelling coefficients *k_sw_* calculated by the approach by Minelli and Doghieri [48], to be used in the NELF model calculations [23,30].

Polymer	Gas	*k*_12_	*k_sw_* (bar^−1^)
PC	CO_2_	0.022	0.00120
	N_2_	−0.018	0.00004
	Ar	0.030	0.00009
	CH_4_	0.035	0.00014
	He	−1.090	≈0
PVC	CO_2_	0.080	0.00070
	N_2_	0.210	≈0
	Ar	0.206	≈0
	CH_4_	0.142	0.00003
PEMA	CO_2_	0.025	0.00210
	N_2_	0.032	0.00003
	Ar	0.061	0.00009
	CH_4_	0.017	0.00024

**Table 3:** Parameters of the STM-GP model: infinite dilution mobility coefficient *L*_10_ and plasticization factor *β*.

Polymer	Gas	*L*_10_ (cm^2^/s)	*β*	Ref.
PC	CO_2_	1.3 × 10^−8^	16.2	[23]
	N_2_	1.2 × 10^−8^	0	[30]
	Ar	2.0 × 10^−8^	0	[30]
	CH_4_	3.9 × 10^−9^	9	[30]
	He	5.2 × 10^−6^	0	[30]
PVC	CO_2_	1.5 × 10^−9^	22	[30]
	N_2_	1.8 × 10^−9^	0	[30]
	Ar	3.1 × 10^−9^	0	[30]
	CH_4_	4.7 × 10^−10^	0	[30]
PEMA	CO_2_	2.5 × 10^−8^	39.5	[23]
	N_2_	4.2 × 10^−8^	0	[30]
	Ar	6.0 × 10^−8^	5	[30]
	CH_4_	1.1 × 10^−8^	10	[30]

**Table 4:** Relative average errors *ε_ave_* among experimental time lag data and model predictions obtained by the present STM-GP model and by the dual mode based approaches (TI: total immobilization; PI: partial immobilization: DDM: dual diffusion).

Polymer	Gas	Relative Average Deviation *ε_ave_*			
		DMS-TI	DMS-PI	DDM	STM-GP
PC	CO_2_	45.2	13.5	11.4	11.0
	Ar	40.4	18.3	11.8	9.3
	CH_4_	92.6	30.0	21.6	12.2
	N_2_	48.6	13.9	10.1	10.8
	He	47.6	9.4	9.6	21.5
PVC	CO_2_	8.2	4.6	17.2	4.3
	Ar	26.9	24.4	18.4	7.4
	CH_4_	39.2	18.6	7.9	5.7
	N_2_	28.3	24.1	16.4	4.8
PEMA	CO_2_	not applicable			17.4
	Ar	5.8	3.7	1.7	6.8
	CH_4_	11.6	6.5	4.7	5.6
	N_2_	10.3	10.3	10.3	4.6

## References

[1] Sanders D.F., Smith Z.P., Guo R., Robeson L.M., McGrath J.E., Paul D.R., Freeman B.D. (2013). Energy-efficient polymeric gas separation membranes for a sustainable future: A review. Polymer.

[2] Baker R.W., Low B.T. (2014). Gas Separation Membrane Materials: A Perspective. Macromolecules.

[3] Li Y., Wang S., He G., Wu H., Pan F., Jiang Z. (2015). Facilitated transport of small molecules and ions for energy-efficient membranes. Chem. Soc. Rev..

[4] Jayakumar A., Sethu S.P., Ramos M., Robertson J., Al-Jumaily A. (2015). A technical review on gas diffusion mechanism and medium of PEM fuel cell. Ionics.

[5] Teo J.T., Mishra A., Park I., Kim Y.J., Park W.T., Yoon Y.J. (2016). Polymeric Biomaterials for Medical Implants and Devices. ACS Biomater. Sci. Eng..

[6] Chu C.S., Lo Y.L., Sung T.W. (2011). Review on recent developments of fluorescent oxygen and carbon dioxide optical fiber sensors. Photonic Sens..

[7] Rai D.R., Paul S. (2007). Packaging requirements of highly respiring produce under modified atmosphere: A review. J. Food Sci. Technol..

[8] Wijmans J.G., Baker R.W. (1995). The solution-diffusion model: A review. J. Membr. Sci..

[9] Crank J. (1975). Mathematics of Diffusion.

[10] Daynes H.A. (1920). The process of diffusion through a rubber membrane. R. Soc. Proc. A.

[11] Michaels A.S., Vieth W.R., Barrie J.A. (1963). Solution of gases in polyethylene terephthalate. J. Appl. Phys..

[12] Michaels S., Vieth W.R., Barrie J.A. (1963). Diffusion of gases in polyethylene terephthalate. J. Appl. Phys..

[13] Vieth W.R., Sladek K.J. (1965). A model for diffusion in a glassy polymer. J. Colloid Sci..

[14] Paul D.R. (1969). Effect of immobilizing adsorption on the diffusion timelag. J. Polym. Sci. A.

[15] Paul D.R., Koros W.J. (1976). Effect of partially immobilizing sorption of permeability and the diffusion timelag. J. Polym. Sci. Polym. Phys. Ed..

[16] Tshudy J.A., Frankenberg C.V. (1973). A model incorporating reversible immobilization for sorption and diffusion in glassy polymers. J. Polym. Sci. Polym. Phys..

[17] Wang L., Corriou J.P., Castel C., Favre E. (2013). Transport of gases in glassy polymers under transient conditions: Limit-behavior investigations of dual-mode sorption theory. Ind. Eng. Chem. Res..

[18] Doghieri F., Biavati D., Sarti G.C. (1996). Solubility and diffusivity of ethanol in PTMSP: Effects of activity and of polymer ageing. Ind. Eng. Chem. Res..

[19] Galizia M., de Angelis M.G., Finkelshtein E., Yampolskii Y.P., Sarti G.C. (2011). Sorption and transport of hydrocarbons and alcohols in addition-type poly(trimethyl silyl norbornene). I: Experimental data. J. Membr. Sci..

[20] Chiou J.S., Paul D.R. (1986). Sorption and transport of CO_2_ in PVF2/PMMA blends. J. Appl. Polym. Sci..

[21] Bos A., Pünt I.G.M., Wessling M., Strathmann H. (1999). CO_2_-induced plasticization phenomena in glassy polymers. J. Membr. Sci..

[22] Bearman R.J., Kirkwood J.G. (1958). Statistical mechanics of transport processes. XI. Equations of transport in multicomponent systems. J. Chem. Phys..

[23] Minelli M., Sarti G.C. (2013). Permeability and diffusivity of CO_2_ in glassy polymers with and without plasticization. J. Membr. Sci..

[24] Doghieri F., Sarti G.C. (1996). Nonequilibrium lattice fluids: A predictive model for the solubility in glassy polymers. Macromolecules.

[25] De Angelis M.G., Sarti G.C. (2011). Solubility of gases and liquids in glassy polymers. Annu. Rev. Chem. Biomol. Eng..

[26] Minelli M., Sarti G.C. (2013). Permeability and solubility of carbon dioxide in different glassy polymer systems with and without plasticization. J. Membr. Sci..

[27] Minelli M. (2014). Modeling CO_2_ solubility and transport in poly(ethylene terephthalate) above and below the glass transition. J. Membr. Sci..

[28] Minelli M., Sarti G.C. (2016). Gas permeability in glassy polymers: A thermodynamic approach. Fluid Phase Equilib..

[29] Minelli M., Sarti G.C. (2017). Thermodynamic modeling of gas transport in glassy polymeric membranes. Membranes.

[30] Minelli M., Sarti G.C. (2015). Thermodynamic model for the permeability of light gases in glassy polymers. AIChE J..

[31] Minelli M., Sarti G.C. (2015). Thermodynamic basis for vapor permeability in Ethyl Cellulose. J. Membr. Sci..

[32] Minelli M., Sarti G.C. (2017). Elementary prediction of gas permeability in glassy polymers. J. Membr. Sci..

[33] Toni E., Minelli M., Sarti G.C. (2018). A predictive model for the permeability of gas mixtures in glassy polymers. Fluid Phase Equilib..

[34] Baschetti M.G., Doghieri F., Sarti G.C. (2001). Solubility in glassy polymers: Correlations through the nonequilibrium lattice fluid model. Ind. Eng. Chem. Res..

[35] Doghieri F., Sarti G.C. (1997). Solubility, diffusivity, and mobility of n-pentane and ethanol in poly (1-trimethylsilyl-1-propyne). J. Polym. Sci. Part B Polym. Phys..

[36] Sarti G.C., de Angelis M.G. (2012). Calculation of the solubility of liquids solutes in glassy polymers. AIChE J..

[37] De Angelis M.G., Sarti G.C. (2008). Solubility and diffusivity of gases in mixed matrix membranes containing hydrophobic fumed silica: Correlations and predictions based on the NELF model. Ind. Eng. Chem. Res..

[38] Minelli M., Campagnoli S., de Angelis M.G., Sarti F.G.C. (2011). Predictive model for the solubility of fluid mixtures in glassy polymers. Macromolecules.

[39] Minelli M., Friess K., Vopička O., de Angelis M.G. (2013). Modeling gas and vapor sorption in a polymer of intrinsic microporosity (PIM-1). Fluid Phase Equilib..

[40] Sanchez C., Lacombe R.H. (1976). An elementary molecular theory of classical fluids. Pure fluids. J. Phys. Chem..

[41] Chapman W.G., Gubbins K.E., Jackson G., Radosz M. (1989). SAFT: Equation-of-state solution model for associating fluids. Fluid Phase Equilib..

[42] Gross J., Sadowski G. (2001). Perturbed-chain SAFT: An equation of state based on a perturbation theory for chain molecules. Ind. Eng. Chem. Res..

[43] Doghieri F., Quinzi M., Rethwisch D., Sarti G.C., Pinnau I., Freeman B.D. (2004). Advanced Materials for Membrane Separations.

[44] Sarti G.C., Doghieri F. (1998). Predictions of the solubility of gases in glassy polymers based on the NELF model. Chem. Eng. Sci..

[45] De Angelis M.G., Sarti G.C., Doghieri F. (2007). NELF model prediction of the infinite dilution gas solubility in glassy polymers. J. Membr. Sci..

[46] Rodgers P.A. (1993). Pressure–volume–temperature relationships for polymeric liquids: A review of equations of state and their characteristic parameters for 56 polymers. J. Appl. Polym. Sci..

[47] Sanchez C., Lacombe R.H. (1978). Statistical thermodynamics of polymer solutions. Macromolecules.

[48] Minelli M., Doghieri F. (2012). A predictive model for vapor solubility and volume dilation in glassy polymers. Ind. Eng. Chem. Res..

[49] Minelli M., Doghieri F. (2014). Predictive model for gas and vapor solubility and swelling in glassy polymers I: Application to different polymer/penetrant systems. Fluid Phase Equilib..

[50] Koros W.J., Paul D.R., Rocha A.A. (1976). Carbon dioxide sorption and transport in polycarbonate. J. Polym. Sci. Polym. Phys. Ed..

[51] Koros W.J., Chan A.H., Paul D.R. (1977). Sorption and transport of various gases in polycarbonate. J. Membr. Sci..

[52] El-Hibri M.J., Paul D.R. (1985). Effects of uniaxial drawing and heat-treatment on gas sorption and transport in PVC. J. Appl. Polym. Sci..

[53] Chiou J.S., Paul D.R. (1989). Gas sorption and permeation in poly(ethyl methacrylate). J. Membr. Sci..

